# Third trimester HbA1c and the association with large-for-gestational-age neonates in women with gestational diabetes

**DOI:** 10.20945/2359-3997000000366

**Published:** 2021-04-29

**Authors:** Liliana Fonseca, Miguel Saraiva, Ana Amado, Sílvia Paredes, Fernando Pichel, Clara Pinto, Joana Vilaverde, Jorge Dores

**Affiliations:** 1 Universitário do Porto Centro Hospitalar Departamento de Endocrinologia Porto Portugal Departamento de Endocrinologia, Centro Hospitalar e Universitário do Porto, Porto, Portugal; 2 Hospital de Braga Departamento de Endocrinologia Braga Portugal Departamento de Endocrinologia, Hospital de Braga, Braga, Portugal; 3 Universitário do Porto Centro Hospitalar Departamento de Nutrição Porto Portugal Departamento de Nutrição, Centro Hospitalar e Universitário do Porto, Porto, Portugal; 4 Centro Materno-Infantil do Norte Departamento de Ginecologia e Obstetrícia Porto Portugal Departamento de Ginecologia e Obstetrícia, Centro Materno-Infantil do Norte, Porto, Portugal

**Keywords:** Third trimester HbA1c, large for gestational age, gestational diabetes mellitus, neonatal complications

## Abstract

**Objective:**

To evaluate the association between HbA1c levels measured in the third trimester and the risk for large for gestational age (LGA) in neonates of mothers affected by gestational diabetes mellitus (GDM). Secondarily, we aimed to identify an ideal cut-off for increased risk of LGA amongst pregnant women with GDM.

**Materials and methods:**

Observational retrospective review of singleton pregnant women with GDM evaluated in a diabetes and pregnancy clinic of a tertiary and academic hospital. From January/2011 to December/2017, 1,085 pregnant women underwent evaluation due to GDM, of which 665 had an HbA1c test in the third trimester. A logistic regression model was performed to evaluate predictors of LGA. A receiver-operating-characteristic (ROC) curve was used to evaluate the predictive ability of third trimester HbA1c for LGA identification.

**Results:**

A total of 1,085 singleton pregnant women were evaluated during the study period, with a mean age of 32.9 ± 5.3 years. In the multivariate analysis, OGTT at 0 minutes (OR: 1.040; CI 95% 1.006-1.076, p = 0.022) and third trimester HbA1c (OR: 4.680; CI 95% 1.210-18.107, p = 0.025) were associated with LGA newborns. Using a ROC curve to evaluate the predictive ability of third trimester HbA1c for LGA identification, the optimal HbA1c cut-off point was 5.4% where the sensitivity was 77.4% and the specificity was 71.7% (AUC 0.782; p < 0.001).

**Conclusions:**

Few studies in the Mediterranean population have evaluated the role of HbA1c in predicting neonatal complications in women with GDM. A third trimester HbA1c > 5.4% was found to have good sensitivity and specificity for identifying the risk of LGA.

## INTRODUCTION

The worldwide prevalence of gestational diabetes mellitus (GDM) has increased (1) and it is nowadays the commonest endocrine pregnancy complication.

In 2008, the Hyperglycemia and Adverse Pregnancy Outcome (HAPO) study was the first large-scale multinational study to show that maternal hyperglycemia between 24-28 weeks was linearly and positively correlated with large-for-gestational-age (LGA) infants, caesarian rate, cord-blood serum C-peptide level, and neonatal hypoglycemia. No glycemic threshold for a greater risk was identified for most outcomes. In fact, GDM is associated with several adverse maternal and fetal outcomes; one of the most worrying is the increased risk for macrosomia and later obesity in the offspring (2–6).

Pregnancies complicated by GDM result in maternal and fetal hyperglycemia. When maternal hyperglycemia occurs, glucose in excess crosses the placenta and reaches the fetal circulation, stimulating fetal insulin secretion. Hyperinsulinemia and glucose excess *in utero* cause insulin-sensitive tissue hypertrophy, promoting accelerated growth that can lead to macrosomia and/or large-for-gestational-age (LGA) neonates (1,4,7,8). GDM severity during pregnancy has been clearly linked with fetal overgrowth (2,5,6,9,10,11), while conversely, HbA1c improvement has been associated with a lower risk of LGA (12). HbA1c, a measure of glycated hemoglobin that serves as an indicator of blood glucose control in the three months prior, can also provide information about metabolic control in pregnancy. During pregnancy, interpretation of HbA1c should take into consideration not only the hemodilution phenomenon but also the existence of a reduced erythrocyte life span, especially in late pregnancy (13,14). Third trimester HbA1c target is not yet defined in women with GDM, nor it is currently used to screen for GDM complications. Given the potentially serious consequences for the mother and the child, there is significant interest in predicting the occurrence of LGA, and its accurate identification holds potential for guiding appropriate management and intervention.

The aim of this study was to evaluate the association between HbA1c levels measured in the third trimester and the risk for LGA in neonates of mothers affected by GDM. Secondarily, we aimed to potentially identify an ideal cut-off for increased risk of LGA newborns amongst pregnant women with GDM.

## MATERIALS AND METHODS

We performed an observational retrospective review of singleton pregnant women with GDM in a Diabetes and Pregnancy Clinic of a tertiary and academic hospital, *Centro Materno-Infantil do Norte, Centro Hospitalar e Universitário do Porto*, Portugal. From January/2011 to December/2017, 1,085 pregnant women underwent evaluation due to GDM, 661 of whom had a measurement of third trimester HbA1c.

Diagnosis and classification of GDM were performed according to the International Association for Diabetes in Pregnancy Study Group (IADPSG) 2010 recommendations: fasting plasma glucose (FPG) ≥ 92 mg/dL (5.1 mmol/L) but < 126 mg/dL (7.0 mmol/L) in first trimester or FPG ≥ 92 mg/dL (5.1 mmol/L) and/or glucose ≥ 180 mg/dL (10.0 mmol/L) and/or ≥ 153 mg/dL (8.5 mmol/L), at 1 h and 2 h, respectively after the ingestion of glucose in the 75 g oral glucose tolerance test (OGTT) performed between weeks 24 and 28 of pregnancy (7). Medical nutrition therapy alone or combined with hypoglycemic drugs was the treatment given to achieve the following therapeutic goals: glucose before meals ≥ 95 mg/dL (≥5.3 mmol/L); glucose 1 h after meals: ≥140 mg/dL (≥7.8 mmol/L), according to national standards (15).

Gestational age was estimated by the last menstrual period and was confirmed or corrected by ultrasonography. Pre-pregnancy BMI was calculated from self–reported pre-pregnancy weight and height. Excess gestational weight gain was defined by the IOM guidelines (16). An infant was classified as LGA if its birth weight was ≥ 90th percentile for gestational age, or small-for-gestational-age (SGA) if its birthweight was < 10^th^ percentile for gestational age, based on the Fenton chart (17). Macrosomia was defined as a newborn weight greater than 4,000 g. Prematurity was defined as delivery occurring before the gestational age of 37 weeks. A composite outcome of neonatal complications was created and included at least one of the following: neonatal respiratory distress, neonatal hypoglycemia, neonatal jaundice (requiring phototherapy), shoulder dystocia, clavicle fracture, Erb's palsy or admission to the neonatal intensive care unit.

Relevant demographic, maternal, and infant data, such as maternal age, obstetric history (parity and previous macrosomia), treatment of GDM, Apgar scores at 1 and 5 minutes, adverse perinatal events and congenital malformations were also recorded. Third trimester HbA1c values were recorded at a median of 34 (IQR: 31-37) weeks of gestation. HbA1c was evaluated using an affinity chromatography method (Variant II turbo, BioRad Laboratories, CA, USA), with intra- and inter-assay coefficients of variation of < 0.78% and < 0.66%.

### Statistical analysis

Statistical analysis was performed using IBM SPSS® version 25.0 and MedCalc®, p-values < 0.05 were considered significant. For continuous quantitative variables, distribution normality was tested through histogram observation and kurtosis and skewness analysis. The results are presented as mean values ± standard-deviation and median values (25-75 percentiles). The chi-square test was used to analyze differences between groups in categorical variables. The Student t-test for independent variables and the Mann-Whitney test were used to compare continuous variables with normal and non-normal distribution between groups, respectively. A logistic regression model was performed to evaluate predictors of LGA, adjusting for potential confounders using a stepwise regression with a forward inclusion approach. A receiver-operating-characteristic (ROC) curve was used to evaluate the predictive ability of third trimester HbA1c for LGA identification.

This study was approved by the local Ethics committee (157-DEFI/156-CES). Due to the retrospective nature of the study, consent to participate was waived by the Ethics Committee.

## RESULTS

A total of 1,085 singleton pregnant women were evaluated during the study period; 85% (n = 922) were Portuguese Caucasian women with a mean age of 32.9 ± 5.3 years. Regarding pre-pregnancy BMI, 34.5% (374/1,085), 34.6% (375/1,085) and 29.5% (320/1,085) of the women had normal weight, overweight, and obesity, respectively. Considering the IOM recommendations for weight gain during pregnancy, 31.5% had an excessive gain. Mean gestational age at delivery was 38.5 ± 1.5 weeks and 7.8% (85/1,085) were preterm. Concerning the 1,085 newborns, mean neonatal birth weight was 3,188.5 ± 49.5 g, 4.5% (49) of them were LGA, 12.4% (134) were SGA using the Fenton chart, but using the International Standards for Size at Birth, the percentage of LGA increases to 6.9% (75) and SGA reduces to 8.9% (97); 4.8% (52) were macrosomic and 19.2% (208) had at least one adverse neonatal outcome. The clinical characteristics of the pregnant women with GDM are presented in Table 1.

**Table 1:** Clinical characteristics of pregnant women with gestational diabetes mellitus

		N = 1085
Age (years)		32.9 ± 5.3
Body mass index (kg/m^2^)		26.5 ± 5.6
Pre-pregnancy BMI category (kg/m^2^)		
	Low weight (<18)	1.5% (16/1085)
	Normal weight (18-24.9)	34.5% (374/1085)
	Overweight (25-29.9)	34.6% (375/1085)
	Obese (≤30)	29.5% (320/1085)
Multiparous		13.9% (151/1085)
Gestation at GDM diagnosis (weeks)*		25 (19-27)
Metformin therapy		5.2% (56/1085)
Insulin therapy		36.3% (395/1085)
Timing of insulin initiation (weeks)*		30 (23-32)
Total daily insulin dose (units)*		23 (14–27)
Gestational weight gain (IOM)		
	Insufficient	35.5% (380/1069)
	Adequate	32.9% (352/1069)
	Excessive	31.5% (337/1069)
Gestational age at delivery (weeks)		38.5 ± 1.5
Prematurity		7.8% (85/1085)
Neonatal birth weight (g)		3188.5 ± 49.5
Small for gestational age (SGA)^1^		12.4% (134/1085)
Large for gestational age (LGA)^1^		4.5% (49/1085)
Macrosomia		4.8% (52/1085)
Apgar score at 1 minutes*		9 (8–9)
Apgar score at 5 minutes*		10 (10–10)
At least one adverse neonatal outcome		19.2% (208/1067)
Neonatal respiratory distress		2.7% (18/671)
Neonatal hypoglycaemia		4.8% (51/1065)
Neonatal jaundice		9.0% (98/1085)
Shoulder dystocia, fractures and Erb's palsy		1.3% (14/1085)
Admission to neonatal intensive care unit		4.5% (49/1085)
Congenital malformations		2.0% (22/1085)
Perinatal death		0.2% (2/1085)

Data are presented as mean ± standard deviation, unless otherwise indicated by

* corresponding to data presented as median, 25th and 75th percentiles. BMI: body mass index; SGA: SMALL for gestational age; LGA: large for gestational age.

1 Fenton chart.

When comparing pregnant women with and without LGA offspring, pregnant women with LGA offspring had a greater prevalence of a previous macrosomic newborn (24.5% vs. 4.4%; p < 0.001), a higher prevalence of pre-pregnancy obesity (49.0% vs. 28.6%, p < 0.002) and a higher excessive gestational weight gain (52.1% vs. 30.6%; p = 0.002). Woman with LGA newborns also presented higher fasting blood glucose in the first trimester [92.0 (83.3-102.8) vs. 85.0 (78.0-94.0), p < 0.001), higher glucose at time 0 in the OGTT [93.5 (86.8-105.0) vs. 85.0 (77.0-92.0); p < 0.001], a higher third trimester HbA1c [5.70 (5.35-5.80) vs. 5.30 (5.00-5.50); p < 0.001] and a higher rate insulin therapy (59.2 vs. 53.3%; p = 0.001). A comparison of the clinical characteristics of pregnant women with and without LGA offspring is presented in Table 2.

**Table 2:** Comparison of the clinical characteristics of pregnant women with and without large for gestational age offspring

		Women with LGA newborns	Women without LGA newborns	p
Maternal age (years)*		35.0 (31.0-37.5)	33 (29.0-37.0)	0.154
Initial BMI (kg/m^2^) *		27.1 (22.5-30.7)	26.6 (22.9-30.4)	<0.001
Pre-pregnancy BMI category				
	Low weight	0%	1.5% (16/1036)	0.785
	Normal weight	20.4% (10/49)	35.1% (364/1036)	0.034
	Overweight	30.6% (15/49)	34.7% (360/1036)	0.552
	Obese	49.0% (24/49)	28.6% (296/1036)	0.002
Gestational weight gain (IOM)				
	Insufficient	18.8% (9/48)	36.3% (371/1021)	0.013
	Adequate	29.2% (14/48)	33.1% (338/1021)	0.570
	Excessive	52.1% (25/48)	30.6% (312/1021)	0.002
Previous macrosomia history		24.5% (12/49)	4.4% (46/1036)	<0.001
First trimester fasting blood glucose (mg/dL)*		92.0 (83.3-102.8)	85.0 (78.0-94.0)	<0.001
OGTT (minutes)*				
	0	93.5 (86.8-105.0)	85.0 (77.0-92.0)	<0.001
	60	190.5 (138.5-214.5)	180.0 (157.0-193.0)	0.174
	120	160.5 (135.5-180.8)	156.0 (129.0-169.0)	0.642
Insulin therapy		59.2% (29/49)	35.3% (366/1036)	0.001
Third trimester HbA1c*		5.70 (5.35-5.80)	5.30 (5.00-5.50)	<0.001

Data are presented as mean ± standard deviation, unless otherwise indicated by

* corresponding to data presented as median, 25th and 75th percentiles. BMI: body mass index; IOM: Institute of Medicine; OGTT: 75 g oral glucose tolerance test.

A logistic regression was performed to identify variables that could predict LGA risk (Table 3). In the univariate analysis, previous macrosomia history, pre-pregnancy obesity, excessive gestational weight gain, higher fasting blood glucose in the 1^st^ trimester and at 0' in the OGTT, need for insulin therapy, and higher third trimester HbA1c were factors associated with LGA. In the multivariate analysis, the following factors were associated with LGA newborns: OGTT at 0 minutes (OR: 1.040; CI 95% 1.006-1.076, p = 0.022) and third trimester HbA1c (OR: 4.680; CI 95% 1.210-18.107, p = 0.025). Excessive gestational weight gain, pre-pregnancy obesity and previous macrosomia history lost statistical significance in the multivariate analysis; nevertheless, they presented a *p* value close to 0.05.

**Table 3:** Predictors of large for gestational age

		Univariate analysis		Multivariate analysis	
		Crude OR (CI 95%)	p	Adjusted OR (CI 95%)	p
Pre-pregnancy BMI category					
	Normal weight	Reference		Reference	
	Overweight	1.500 (0.665-3.383)	0.329	1.306 (0.272-6.264)	0.738
	Obese	2.778 (1.308-5.900)	0.008	4.733 (0.905-24.765)	0.066
Excessive gestational weight gain (IOM)		2.464 (1.377-4.409)	0.002	3.028 (0.862-10.640)	0.084
Previous macrosomia history		6.180(2.968-12.866)	<0.001	6.130 (0.986-38.123)	0.052
Fasting blood glucose (mg/dL)		1.057 (1.032-1.083)	<0.001	1.021 (0.955-1.090)	0.535
OGTT (0 minutes)		1.030 (1.14-1.047)	<0.001	1.040(1.006-1.076)	0.022
Insulin therapy		2.657 (1.482-4.764)	0.001	1.319 (0.387-4.492)	0.658
Third trimester HbA1c		13.606 (6.061-30.544)	<0.001	4.680 (1.210-18.107)	0.033

IOM: Institute of Medicine; OGTT: 75 g oral glucose tolerance test.

**Figure 1:**
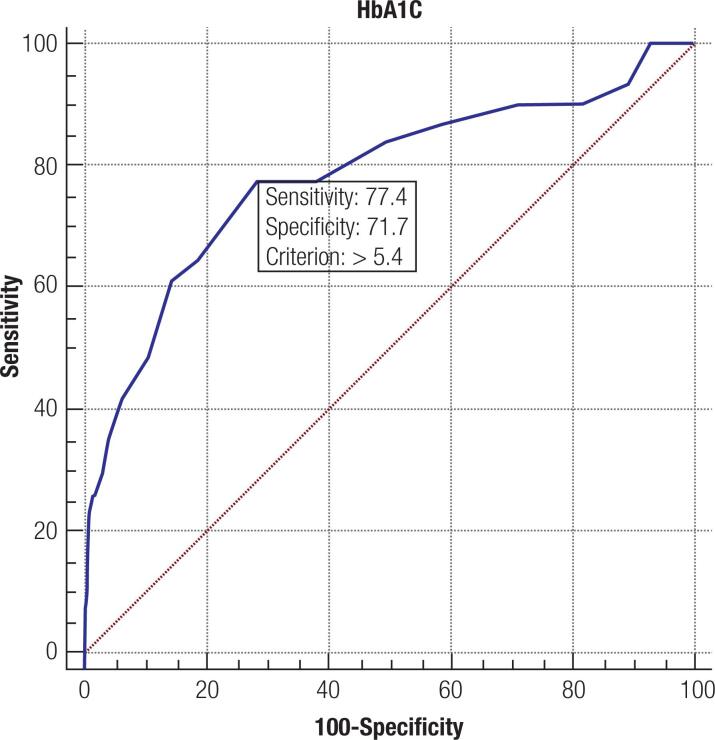
ROC curve analysis of third trimester HbA1c values for LGA prediction using the Fenton Chart.

**Figure 2:**
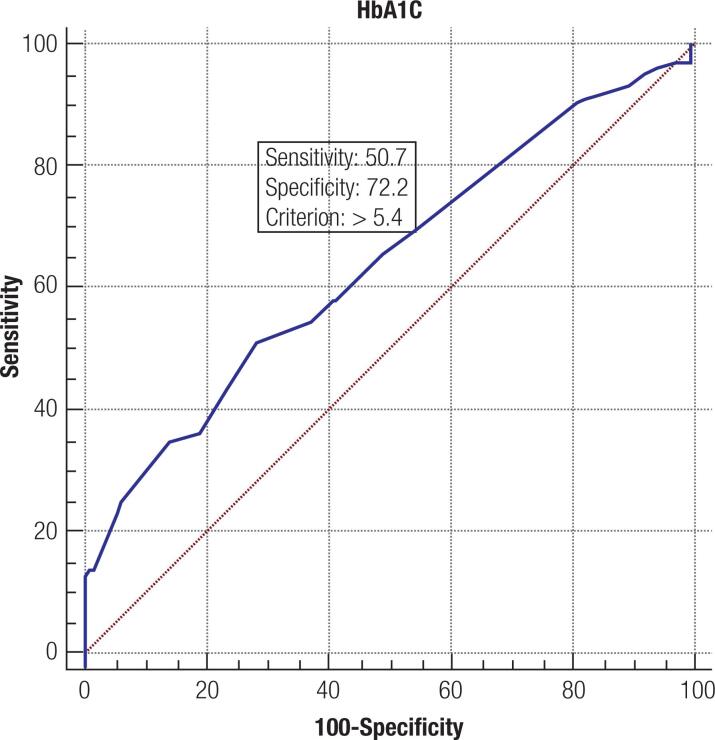
ROC curve analysis of third trimester HbA1c values for LGA prediction using the International Standards for Size at Birth.

Using a ROC curve to evaluate the predictive ability of third trimester HbA1c for LGA identification, the optimal HbA1c cut-off point was at 5.4% (36 mmol/mol) where the sensitivity was 77.4% and the specificity was 71.7% (AUC 0.782; p < 0.001) using the Fenton Chart to define LGA (Figure 1). Using International Standards for Size at Birth to define LGA, the optimal HbA1c cut-off point remains at 5.4% (36 mmol/mol) where the sensitivity was 50.7% and the specificity was 72.2% (AUC 0.638; p < 0.001) (Figure 2).

## DISCUSSION

In our study, in a seven-year cohort of 1085 pregnancies, the incidence of LGA newborns was 4.5% using the Fenton chart and 6.9% using the International Standards for Size at Birth. In the literature, the overall LGA incidence varies from 5%-20% in developed countries (18). It is a known fact that poorly controlled GDM, pre-pregnancy obesity and excessive weight gain during pregnancy increase the risk of LGA and several other neonatal complications (2,11,19–21). Several studies have reported that previous macrossomia history, pre-pregnancy obesity and weight gain during pregnancy above the recommended guidelines are associated with an increased incidence of LGA (22–26). Dong and cols. (2018) described that the LGA offspring is characterized by decreased fetal insulin sensitivity and impaired β-cell function (27). Moreover, in this study, pregnant women with characteristics that favored the development of insulin resistance, such as pre-pregnancy obesity, higher weight gain and need for insulin therapy, were associated with a higher risk of having LGA newborns. Interestingly, the prevalence of macrossomia was slightly higher than that of LGA newborns (three more cases). A possible explanation for this finding is that gestational age at birth in our study was later, with a mean of 38.5 weeks, and macrossomia is defined as birth weight > 4,000 g, irrespective of gestational age. This is contrary to the definition of LGA that correlates birth weight with gestational age. Moll and cols., in their study, also describes a higher prevalence of macrosomia compared to LGA (28).

In our logistic regression model, pre-pregnancy obesity, excessive gestational weight gain (IOM) and previous macrosomia history lost significance as LGA predictors, probably because of insufficient power due to sample size. Nevertheless, glucose levels at the beginning of the OGTT and third trimester HbA1c remained as independent factors for predicting the risk of having LGA offspring.

Tavares and cols. (26) described that LGA newborns were significantly more common in the group of women with combined change in the OGTT (hyperglycemia both in fasting and after a dextrose load), even after adjustment for potential confounders. Brankica and cols. (29) and Ouzilleau and cols. (30) found that high levels of fasting blood glucose in the OGTT were better predictors of LGA. Mello and cols. (31) reported fasting [1.04 (CI 95% 1.01-1.06)] and 1 h [1.03 (CI 95% 1.02-1.03)] and 2 h [1.03 (1.02-1.04)] glucose values in the OGTT performed between 26-30 weeks of gestation as independent risks factors for LGA newborns. In our study, only fasting blood glucose in the OGTT was found to be an independent risk factor for LGA newborns, with an odds ratio similar to that reported by Mello and cols. (31). Impaired fasting glucose (IFG) and impaired glucose tolerance (IGT) represent two different types of glucose metabolism disorder. In fact, Tripathy and cols. (32) showed that not only do IGT and IFG have poor concordance between them, but also that they have different underlying pathophysiological mechanisms. The major determinant of IFG seems to be defective insulin action rather than an impaired β-cell function: the fasting glucose level is largely determined by endogenous glucose production, which depends on hepatic insulin sensitivity. As such, subjects with defective hepatic insulin sensitivity are more prone to developing IFG. Moreover, IFG seems to be more closely related to other features of metabolic syndrome (elevated triglyceride and total cholesterol concentrations, lower HDL cholesterol concentrations and higher waist-to-hip ratio) than IGT; thus, patients with IFG are at higher risk of developing diabetes (32). On the other hand, IGT seems to be mainly determined by impaired insulin secretion (β-cell dysfunction) in relation to glycemia and the degree of insulin resistance (32).

HbA1c has been proposed as a useful marker for predicting LGA and other neonatal complications. During pregnancy, the life span of red blood cells decreases from about 120 days to about 90 days, together with increased erythropoietin production. HbA1c values also decrease by 12-16 weeks of gestation, with a further decrease that plateaus by gestational weeks 20-24. HbA1c levels may start to rise again in the third trimester (33–36). The findings of this study identify third trimester HbA1c level as a determinant of LGA in pregnancies affected with GDM. We found that for every 1%-unit increase in third trimester HbA1c, the odds of having an LGA infant are increased by a factor of 4.7 (CI 95% 1.2-18.1, p = 0.025).

Shushan and cols. found that early control of GDM (before 34 weeks) resulted in a an 18% lower rate of LGA infants, compared to late control of GDM (after 34 weeks) (37). This is a reminder that GDM needs to be well controlled to ensure better fetal outcomes, identifying the risk of a large-for-gestational age fetus before the 34th week, as this will enable measures to be implemented for better glycemic control and decrease the risk for a LGA fetus.

Mañé and cols. (2019) have shown that in Latin-Americans, a first trimester HbA1c ≥ 5.8% (40 mmol/mol) and HbA1c ≥ 5.9% (41 mmol/mol) were associated with a higher risk of macrosomia and LGA, respectively. On the other hand, in a South-Central Asian population, the Hb1Ac cut-off associated with macrosomia and LGA was a first trimester HbA1c ≥ 5.7% (39 mmol/mol) and ≥ 5.4% (36 mmol/mol), respectively. Curiously, no association was found between first trimester HbA1c and obstetric outcomes among Caucasians (38). Sweeting and cols. suggested that a single HbA1c measurement during the time of universal screening for GDM at 24-28 weeks of gestation can be useful in clinical practice, to classify women with GDM with high or low risk for a LGA infant. They found that a HbA1c > 5.9% (41 mmol/mol) at 24-28 weeks of gestation appears to be associated with a higher risk of adverse outcomes, which included macrosomia, LGA, cesarean section and hypertensive disorders (39). Wong and cols. (2017) showed that elevated HbA1c, measured at diagnosis of GDM or at 36 weeks of gestation, were both independent predictors of LGA offspring and neonatal hypoglycaemia. They reported HbA1c cut-offs of 5.4% (36 mmol/mol) at diagnosis and 5.5% (37 mmol/mol) at 36 weeks (40). Our results also evidence that women with GDM who had a third trimester HbA1c, between 31-37 weeks of gestation (median of 34 weeks), above 5.4% (36 mmol/mol) were more likely to have an LGA infant. Our results are in accordance with those of other studies that found that HbA1c could be helpful in predicting LGA. For example, Barquiel and cols. found that a third trimester HbA1c level > 5% (31 mmol/mol) is a modifiable risk factor—that influences neonatal overgrowth and neonatal complications in mothers with GDM (22). There are few reports about the definition of a cut-off value of HbA1c in gestational diabetes, and those that do exist vary in relation to mother's ethnicity, trimester of evaluation and method of definition.

This study has some limitations that deserve comment. First, it was a retrospective study with an associated bias that cannot be ruled out. Second, the majority of our population is Caucasian and from the Mediterranean area; therefore, our results are not generalizable to other populations. This study also has some strengths; we have a large sample size of women with GDM, all evaluated by the same protocol. Moreover, the identification of a third trimester A1c cut-off for LGA could help physicians in clinical practice.

In conclusion, in this study, a third trimester HbA1c > 5.4% (36 mmol/mol) was found to have good sensitivity and specificity for the identification of an increased risk of LGA offspring among women with GDM. This highlights the importance and value of a HbA1c measurement in the third trimester, as it can alert physicians to this risk and help them to better manage these pregnant women. Few studies have been published in the Mediterranean population concerning the definition of a third trimester HbA1c value from which the risk of LGA increases, and further studies are needed, to validate the HbA1c cut-off point suggested by our study.
